# Characterization of phenolic components of black teas of different origins and the effect of brewing duration on quality properties

**DOI:** 10.1002/fsn3.3782

**Published:** 2023-10-30

**Authors:** Mehmet Emin Aydemir, Kasım Takım, Mustafa Abdullah Yılmaz

**Affiliations:** ^1^ Department of Basic Sciences of Veterinary Medicine, Faculty of Veterinary Medicine Harran University Şanlıurfa Turkey; ^2^ Department of Veterinary Food Hygiene and Technology, Faculty of Veterinary Medicine Harran University Şanlıurfa Turkey; ^3^ Department of Pharmaceutical Chemistry, Faculty of Pharmacy Dicle University Diyarbakır Turkey

**Keywords:** antioxidant activity, black tea, brewing time, LC–MS/MS, tea origin

## Abstract

This research aims to identify the phytochemical constituents of 79 different samples of black tea, including varieties from India, Iran (IrT), Turkey (TT), and Sri Lanka. In addition, this study investigates the effect of varying brewing times on the quality characteristics of tea. Therefore, we analyzed the phytochemical content of tea using a novel LC–MS/MS method that we developed, which identifies 53 different phenolic compounds. Furthermore, objective evaluations were conducted on the total phenolic compound, total flavonoid compound, antioxidant activity, and color values at 15, 30, and 60‐min brewing intervals. The prevailing phenolic compounds discovered in the corresponding tea classifications were quantitatively analyzed to be quinic acid, epicatechin gallate, epigallocatechin gallate, epicatechin, epigallocatechin, gallic acid, nicotiflorine, and isoquercitrin. The study found that the TT and IrT groups had the richest phytochemical content and the highest antioxidant activity. The Turkish tea group had the highest measurement for the desired red color, which is considered a sensory property. Infusion color, antioxidant activity, and total phenolic and flavonoid contents showed significant increases with prolonged brewing time. It was important to note that the chemical composition of tea varies according to its origin and brewing conditions. Extending the brewing time improved the quality of the tea. It should be noted, however, that longer brewing times result in a more intense release of flavonoids, and this increase may have a pro‐oxidant effect.

## Graphical abstract

This study aimed to determine the phytochemical constituents of a total of 79 samples of black tea, such as Indian, Iranian (IrT), Turkish (TT), and Sri Lankan varieties. Moreover, investigated how different brewing times would affect the quality characteristics (total phenolic, total flavonoid, antioxidant capacity and color) of tea. As a result of our study, the phytochemical compounds of black tea originating from Turkey, India, Iranian, and Sri Lanka were identified with the 53‐item phenolic compound method, which was the most advanced method in the field and was validated by us. It was also determined how the quality characteristics of these tea groups changed at different brewing times.

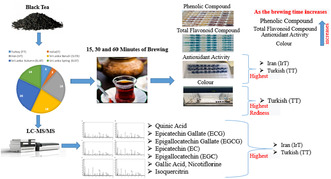

## 1 INTRODUCTION

Tea, derived from the leaves of the *Camellia sinensis* plant native to tropical and subtropical regions, is the second most consumed beverage worldwide after water. With a history spanning over 500 years, it is an economically significant commodity. (Takım & Aydemir, 2018; Üstün & Demirci, 2013) Approximately two‐thirds of the global population partakes in drinking tea. Tea is believed to have originated in China and India, with its homeland located in Assam (Takım & Aydemir, 2021; Üstün & Demirci, 2013). Although the tea plant is generally cultivated in China, India, Sri Lanka, Kenya, Vietnam, Indonesia, Russia, Japan, Myanmar, Turkey, Bangladesh, Iran, Argentina, Uganda, Tanzania, Malawi, Thailand, Nepal, Rwanda, Burundi, and Ethiopia, tea production is chiefly centered in nations like India, China, Sri Lanka, Indonesia, Kenya, and Japan. (Amirahmadi et al., 2013; Kurt & Hacıoğlu, 2013) If tea leaves undergo various processing methods, they can yield different types of tea with diverse appearances, tastes, and flavors, such as black, green, and oolong tea. Black tea is formed by the polyphenol oxidase enzyme that catalyzes oxidation after crushing the tea leaves. The most significant compounds distinguishing these tea groups from other herbal teas are the catechins and caffeine in their composition (Akbay, 2021; Balcı & Özdemir, 2016).

The tea plant exhibits antioxidative, antimicrobial, anti‐cancer, and anti‐inflammatory effects on human health. It has been shown to mitigate cholesterol and blood pressure and minimize the risk of cardiovascular disease. These health benefits arise from its polyphenol compounds and their antioxidant properties. (Yan et al., 2020; Zhang et al., 2019). Typical compounds found in black tea include cotans, theaflavins (De & Ray, 2022), thearubigines, amino acids (Cheng et al., 2023), and alkaloids. The catechins and their polymerized products are the main components of black tea's polyphenolic compounds. Determining the compounds and quantities of the tea is crucial, as the biological activity and drink of the tea are linked to its chemical profile (Pan et al., 2013). Additionally, each type of tea is composed of distinct chemical components (Yang & Liu, 2013). The chemical composition of tea leaves varies based on their genetics, climate, soil structure, and other environmental factors (Takım, 2020). Additionally, changes in the biochemical structure of tea are caused by production methods and standards in the collection and processing phases (Mathew & Abraham, 2006).

The chemical composition of tea can vary based on various factors, such as the particle size of the tea leaves and brewing conditions (time, temperature, amount, number of infusions, and exposure to light). (Pastoriza et al., 2017).

Color is a crucial factor for consumers when deciding on food choices. This is due to the impact of color on taste perception and product quality. In the case of tea, color is particularly important for determining quality characteristics and pricing. The flavanol compounds of black tea are oxidized during fermentation, creating theaflavins, thearubigins, and other polymerization products that significantly affect tea's brightness, vitality, and color (Takım, 2020; Yang & Liu, 2013). Therefore, the color that determines the quality of tea is directly related to the chemical composition of tea (Mathew & Abraham, 2006; Pastoriza et al., 2017).

There are many studies in the literature about the effect of infusion time (Fernando & Soysa, 2015a; Kyle et al., 2007; Ouyang et al., 2017) of black tea on phenolic and flavonoid contents and antioxidant capacity. Nonetheless, these studies are generally not directly applicable to the infusion times commonly used by individuals when brewing tea, namely 3, 6, and 10 min. This is because people typically steep tea for at least 15 min before consuming it, typically no earlier than an hour after brewing. What occurs to the tea during this period interval? Is there an increase or decrease in the phenolic content and antioxidant capacity? Objective answers to these inquiries are expected.

The objective of the study is to identify and analyze the phytochemical composition of 79 samples of black tea, including Turkish, Indian, Iranian, Sri Lankan barudi, Sri Lankan autumn, and Sri Lankan spring black tea, which are grown in various countries and are popularly consumed in the Turkish market. Our newly developed LC–MS/MS method is used to detect the 53 phenolic compounds, total phenolic, total flavonoid, antioxidant capacity, and color characteristics of the samples. The study aims to determine the quality of the tea and the reasons behind its popularity without making subjective evaluations. Additionally, the aim is to investigate the impact of varying brewing durations on the overall phenolic and flavonoid content, antioxidant capacity, and color attributes of tea.

## 2 MATERIALS AND METHODS

### 2.1 Collection and preparation of tea samples for analysis

Tea samples were collected from both imported and domestic black tea sold in the provinces of South‐eastern Anatolia and Eastern Anatolia in Turkey. The number of samples was determined based on the consumption frequency and market share rates of the respective countries. A total of seventy‐nine samples were collected. Samples from six countries were included in the analysis: 6 from Turkey (TT), 3 from India (IT), 18 from Iran (IrT), 18 from Sri Lanka Barudi (SLTB), 20 from Sri Lanka Autumn (SLAT), and 14 from Sri Lanka Spring (SLST). To ensure homogeneity, teas with the same origin were mixed prior to analysis.

Following the determination of 53 phenolic compounds in 6 tea groups through the LC–MS/MS method, each tea group was brewed for varying durations (15, 30, and 60 min) and analyzed for total phenolic, total flavonoid content, antioxidant capacities, and color. Six samples were collected from Turkey (TT), India (IT), Iran (IrT), and Sri Lanka Barudi (SLTB), Sri Lanka Autumn (SLAT), and Sri Lanka Spring (SLST). The tea sample was infused using a ratio of 1:5 (1 g of sample to 5 mL of water (100°C). While determining the brewing times and tea brewing rates, the rate and time most preferred by the people in Turkey were used. The analysis procedure was repeated three times to ensure the accuracy and reliability of the results.

Preparation of plant extracts for LC–MS/MS: The tea samples were first kept in an etüv (**Nüve KD 200, Türkiye)** at 70°C for 4 h to allow the moisture to evaporate. Then the dried materials (10 g) of the tea samples were pulverized. These powdered materials were extracted 3 times by the maceration method with water (6 h) at ambient temperature. Water was chosen as extraction solvent because it is the ideal solvent for the extraction of the studied compounds (aglycone and glycosidic forms of phenolic compounds), and local people use these teas by extracting them with water. After filtration of the extracts, the solvents were evaporated using a rotary evaporator at 30°C under vacuum. Finally, water extracts of teas of different origins were obtained for analysis.

### 2.2 Identification and quantification of tea phenolic compounds LC–MS/MS

LC–MS/MS analysis was carried out at Dicle University Central Research Laboratory. The analytical method used in this study was developed by Yilmaz (2020). This method has been used and validated for many plants, especially Jerusalem thorn fruits (Takım, 2021). Reference phytochemical standards (consisting of 53 phytochemicals) were acquired from Sigma‐Aldrich (Steinheim, Germany). Internal standards, including ferulic acid‐D3, rutin‐D3, and quercetin‐D3, as well as 1,5‐dicaffeoylquinic acid, were purchased from TRC (Toronto, Canada). The study employed a LC–MS/MS method to analyze the phytochemicals, including quinic acid, fumaric acid, gallic acid, malic acid, epigallocatechin (EGC), protocatechuic acid, catechin, gentisic acid, chlorogenic acid, and protocatechuic aldehyde. The following plant compounds were identified in the sample: tannic acid, epigallocatechin gallate (EGCG), 4‐OH benzoic acid, epicatechin (EC), vanillic acid, caffeic acid, syringic acid, vanillin, syringic aldehyde, daidzin, epicatechin gallate (ECG), piceid, ferulic acid, p‐coumaric acid, sinapic acid, coumarin, salicylic acid, cynaroside, miquelianin, rutin, isoquercitrin, hesperidin, o‐coumaric acid, genistin, rosmarinic acid, ellagic acid, cosmosiin, quercitrin, astragalin, and nicotiflorin. Fisetin, cynarine, daidzein, quercetin, naringenin, luteolin, hesperetin, genistein, apigenin, kaempferol, amentoflavone, chrysin, and acacetin were utilized to examine the phenolic compounds in tea samples. Three isotopically labeled internal standards (quercetin D3, rutin D3, and ferulic acid D3) were employed for flavonoids, flavonoid glycosides, and non‐flavonoid compounds, respectively, to compensate for the matrix effects and analyte losses during sample preparation and analyses, thereby enhancing the credibility of the results. In the investigation of phenolic components, this study examined water extracts prepared using both decoction and infusion methods, as well as methanol and ethanol extracts. The LC–MS/MS system employed a Shimadzu Nexera UHPLC device and a Shimadzu 8040 triple quadrupole mass spectrometer. The liquid chromatography system included a gradient pump, degasser, column oven, and autosampler models LC‐30 AD, DGU‐20A3R, CTO‐10ASvp, and SIL‐30 AC, respectively. Chromatographic separation was conducted on an Inertsil ODS‐4 C18 column (100 × 2.1 mm, 2 μm) with an elution gradient consisting of eluent A (water +5 mM ammonium formate +0.1% formic acid) and eluent B (methanol +5 mM ammonium formate +0.1% formic acid). Chromatographic separation was conducted on an Inertsil ODS‐4 C18 column (100 × 2.1 mm, 2 μm) with an elution gradient consisting of eluent A (water +5 mM ammonium formate +0.1% formic acid) and eluent B (methanol +5 mM ammonium formate +0.1% formic acid). Additionally, the water phase was supplemented with 10 mM ammonium formate and 0.1% formic acid to enhance the chromatographic separation and ionization. The gradient elution profile used was 20%–100% B for 0–25 min, 100% B for 25–35 min, and 20% B for 35–45 min. The solvent flow rate and injection volume were fixed at 0.5 mL/min and 5 μL, respectively. The mobile phase flow rate was set to 0.25 mL/min, and the injection volume was 4 μL. The triple quadrupole mass spectrometer featured an ESI (electrospray ionization) source, operating in negative and positive modes. The collected LC‐ESI‐MS/MS data have been processed through registered LabSolutions software (Shi‐madzu, Kyoto, Japan). The analytes were quantitatively analyzed in MRM (multiple reaction monitoring) mode, combining parent ions with one or two cleavage products. The mass spectrometer was optimized for several parameters, including: interface temperature at 350°C, DL temperature at 250°C, heat block temperature at 400°C, nebulizer gas (N2) flow at 3 L/min, and drying gas temperature at 15 L/minAfter incorporating standard deviation values into the LC–MS/MS analysis, the results were computed using the subsequent equation. (Standard deviation (±) value of the results = Analyte value result × U value (Relative standard uncertainty at 95% confidence level)/100).

### 2.3 Determination of the antioxidant capacities of tea

#### 2.3.1 ABTS radical scavenging activity assay

The ABTS assay was performed following Necip et al. (Necip et al., 2021). The percentage of radical scavenging activity in the samples was assessed using the ABTS method. The antioxidant capacity was quantified in terms of Trolox.

#### 2.3.2 DPPH radical scavenging activity assay

The determination of DPPH radical scavenging activity was carried out as per Takim and Isik's study (Takım & Işık, 2020). % Radical capture was evaluated using the DPPH method applied to the samples, and antioxidant capacity was expressed in Trolox.

#### 2.3.3 Determination of Total phenolic compounds in tea

The total phenolic content was measured using the Folin–Ciocalteu method (Pereira et al., 2014). High absorbance values indicate high phenolic content. 40 μL of leaf extracts were mixed with 200 μL of Folin–Ciocalteu reagent to form 1 mg/mL. After adding 1160 μL of distilled water, 600 μL of 20% sodium carbonate (Na2CO3) was added 3 min later. The resulting mixture was swirled at room temperature for 2 h. Absorbance was measured at a wavelength of 765 nm using gallic acid as the standard. The antioxidant capacity was subsequently computed by multiplying the required dilution factors with the mg of Gallic acid equivalent per gram of tea.

#### 2.3.4 Determination of Total flavonoid compounds in tea

The total flavonoid content in the water extract of tea groups was determined using the aluminum chloride colorimetric assay to measure antioxidant activity (Marinova et al., 2005). The extract or standard solution of quercetin (at concentrations of 50, 100, 200, 400, and 800 mg LG1) was dispensed into a tube before adding 4 mL of distilled water. The reaction was then initiated by adding 0.3 mL of a 5% NaNO2 solution, followed by the addition of 0.3 mL of a 10% AlCl3 solution after 5 min. At the sixth minute, 2 mL of a 1 M NaOH solution was added, and the total volume was made up to 10 mL with pure water before the solution was thoroughly mixed. The reaction was incubated at room temperature for 60 min. The absorbance against the prepared reagent blank was measured at 510 nm using a UV‐spectrometer. Subsequently, the total flavonoid content of the extract was quantified and expressed as milligrams of quercetin acid equivalents per gram.

#### 2.3.5 Color analysis of tea

The tea samples’ color characteristics were evaluated using the Hunter L* (whiteness/darkness), a* (redness/greenness), and b* (yellowness/blueness) parameters, which were adjusted to a standard observer angle of 10° and D‐65 illumination type. The Chroma Meter (model CR‐5, Konica Minolta, Osaka, Japan) was used to measure the color. To perform the measurements, 5 mL of tea sample was placed in a rectangular glass cell (50 × 38) while maintaining its temperature. Prior to analysis, the instrument's standardization was conducted using a black plate (Mao et al., 2021).

### 2.4 Statistical analysis

The statistical analysis involved evaluating L*, a*, and b* values, total phenolic and total flavonoid content, as well as the antioxidant capacities of the samples. Statistical tests, including Analysis of Variance (ANOVA) and the Duncan test, were conducted using the SPSS version 21.0 package program to compare tea groups and brewing times. Correlation coefficients were determined through Pearson correlation analysis (IBM SPSS, 2012). Statistical analysis of antioxidant activity studies was performed using the GraphPad Prism 5.01 program. Data comparison was carried out using the Two‐Way ANOVA Bonferroni post‐test. Group averages were presented as ± standard deviation and between‐group comparisons as ± standard error. The significance of between‐group differences was assessed through the Bonferroni post‐test. Statistical significance was considered at a significance level of *p* < .05 in all group comparisons, with any significant differences indicated by letters in the accompanying tables. The relationship between tea activity (DPPH, ABTS, TPC, and TFC) and color values (L*, a*, and b*) was analyzed using Principal Component Analysis (PCA) for various brewing times. Minitab 16.2.1 statistical software by MINITAB Inc. (2010) was employed in conducting the PCA statistical analysis. Prior to PCA, all variables were standardized with a zero mean and unit variance.

## 3 RESULTS

### 3.1 Phenolic component results

**FIGURE 1:**
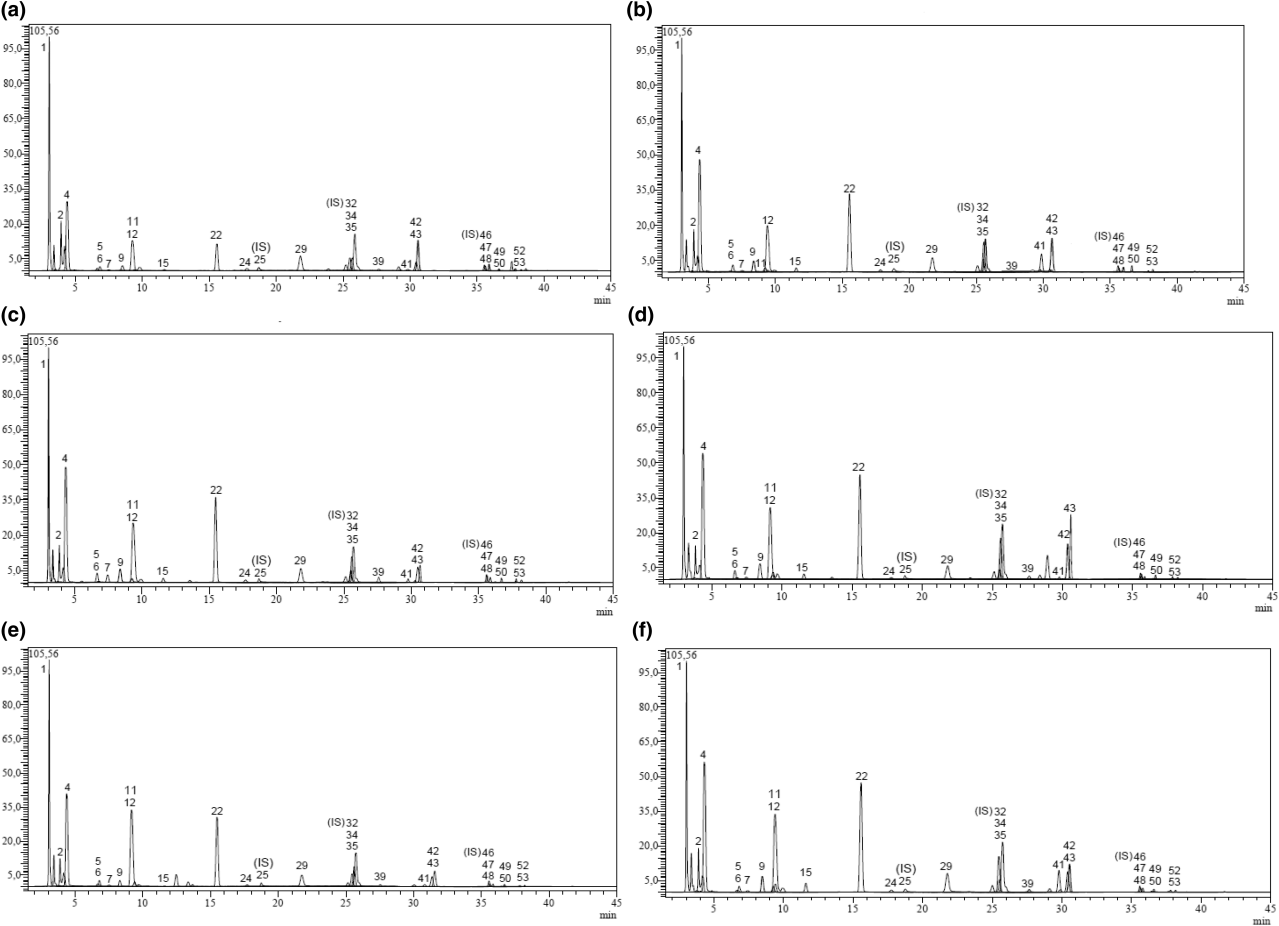
Tea LC–MS/MS chromatograms (a: Turkey Tea, b: İndia Tea, c: Iran Tea, d: Sri Lanka Tea Barudi, e: Sri Lanka Autumn Tea, f: Sri Lanka Spring Tea).

Phenolic component analysis has been conducted on samples of *Camellia sinensis* tea, and this has been documented in literature (Magdalena et al., 2018). This investigation is groundbreaking in its exploration of the most phenolic components (53 phenolics) in a singular tea sample using a singular method, alongside analysis and comparison of tea originating from various countries with significant participation in the world tea market. Tea cultivation occurs in approximately 40 countries worldwide. Our investigation prioritized tea samples from four of the foremost countries in terms of production. LC–MS/MS analysis findings of the tea samples are presented in Table 2 and Figure 1. The results have been calculated based on the dry weight. Table S1 includes the analytical method validation parameters and the standard mixture of LC–MS/MS chromatograms. Table 1 displays the quantitative outcomes of phenolic compounds found in the tea samples, and their peaks are shown in Figure 1.

**TABLE 1:** Identification and quantification of phenolic compounds in different teas (μg Analyte/g tea samples ± standard deviation).

Analyte	TT	IT	IrT	SLTB	SLAT	SLST
Hesperidin	0.476 ± 0.00015	0.547 ± 0.0002	0.698 ± 0.0002	0.389 ± 0.00003	0.364 ± 0.0001	0.337 ± 0.0001
**Quinic acid**	**6.15 ± 0.0022**	**7746 ± 0.003**	**8.92 ± 0.003**	**7323 ± 0.003**	**7855 ± 0.003**	**6508 ± 0.002**
Fumaric acid	0.167 ± 0.00015	0.198 ± 0.00002	0.211 ± 0.0002	0.245 ± 0.00002	0.322 ± 0.00003	0.123 ± 0.00001
**Gallic acid**	**1587 ± 0.02**	**1632 ± 0.002**	**2059 ± 0.012**	**1584** ± **0.002**	**0.913 ± 0.001**	**1185 ± 0.0013**
Protocatechuic acid	0.07 ± 0.0003	0.097 ± 0.00004	0.143 ± 0.00006	**0.126 ± 0.00005**	0.053 ± 0.00002	0.073 ± 0.00003
**Epigallocatechin**	**1796 ± 0.003**	**1033 ± 0.0002**	**1675 ± 0.0002**	**1141 ± 0.0002**	**0.566 ± 0.00008**	**2268 ± 0.00033**
Catechin	0.156 ± 0.0004	0.131 ± 0.00003	0.155 ± 0.00003	0.108 ± 0.00002	0.012 ± 0.00003	0.149 ± 0.000032
Chlorogenic acid	0.125 ± 0.0003	0.096 ± 0.00002	0.158 ± 0.00003	0.113 ± 0.00002	0.005 ± 0.000001	0.094 ± 0.00002
Tannic acid	0.07 ± 0.0001	0.041 ± 0.000007	0.078 ± 0.00001	0.069 ± 0.00001	0.044 ± 0.000008	0.036 ± 0.00007
**Epigallocatechin gallate**	**3865 ± 0.0006**	**2729 ± 0.0004**	**4376 ± 0.0006**	**3.27 ± 0.0005**	**1492 ± 0.0002**	**3695 ± 0.00054**
**Epicatechin**	**4841 ± 0.001**	**2586 ± 0.0006**	**3747 ± 0.0008**	**1346 ± 0.0004**	**0.579 ± 0.00001**	**3968 ± 0.00087**
**Epicatechin gallate**	**4393 ± 0.001**	**3701 ± 0.0008**	**5578 ± 0.012**	**3651 ± 0.0008**	**1087 ± 0.00002**	**2963 ± 0.00067**
p‐Coumaric acid	0.029 ± 0.0006	0.033 ± 0.0006	0.032 ± 0.0006	0.036 ± 0.0007	0.034 ± 0.0006	0.024 ± 0.00005
Salicylic acid	0.038 ± 0.000006	0.035 ± 0.000005	0.037 ± 0.000006	0.031 ± 0.000005	0.032 ± 0.00005	0.024 ± 0.000004
Isoquercitrin	**0.693 ± 0.00015**	**0.721 ± 0.00015**	**1.13 ± 0.00024**	**0.578 ± 0.00012**	**0.325 ± 0.0007**	**0.417 ± 0.00009**
Ellagic acid	0.049 ± 0.000017	0.051 ± 0.000018	0.071 ± 0.000025	0.033 ± 0.000012	0.01 ± 0.000004	0.023 ± 0.000008
Quercitrin	0.026 ± 0.000007	0.428 ± 0.00011	0.33 ± 0.00008	0.902 ± 0.00024	0.32 ± 0.00008	0.42 ± 0.000011
Astragalin	0.361 ± 0.00004	0.257 ± 0.00003	0.578 ± 0.00006	0.322 ± 0.000036	0.162 ± 0.00002	0.178 ± 0.00002
Nicotiflorin	**1.17 ± 0.000012**	**0.63 ± 0.00068**	**1.57 ± 0.00016**	**0.85 ± 0.00009**	**0.58 ± 0.00062**	**0.53 ± 0.00006**
Quercetin	0.041 ± 0.000007	0.044 ± 0.000007	0.068 ± 0.00001	0.051 ± 0.000009	0.049 ± 0.000008	0.029 ± 0.000005
Luteolin	0.003 ± 0.0000009	0.007 ± 0.000002	0.005 ± 0.000002	0.005 ± 0.000002	0.01 ± 0.000003	0.002 ± 0.0000006
Hesperetin	0.003 ± 0.0000009	0.003 ± 0.0000009	0.005 ± 0.0000002	0.004 ± 0.000001	0.004 ± 0.000002	0.002 ± 0.0000006
Naringenin	0.006 ± 0.000002	0.009 ± 0.000004	0.005 ± 0.000002	0.01 ± 0.000004	0.009 ± 0.000004	0.004 ± 0.000001
Kaempferol	0.012 ± 0.0000025	0.01 ± 0.000002	0.021 ± 0.000004	0.019 ± 0.000004	0.038 ± 0.000008	0.006 ± 0.000001
Apigenin	0.001 ± 0.0000002	0.002 ± 0.0000004	0.002 ± 0.000004	0.003 ± 0.0000005	0.005 ± 0.0000009	0.001 ± 0.0000002

*Note*: Analytical method validation parameters (retention time, ion mode, equation, *LOD*/*LOQ, RSD%, linearity range, and recovery (%)* that belong to the LC–MS/MS method) are given in the Table S1, highly abundant compounds in teas and their amounts are highlighted in bold.

Abbreviations: IrT, Iran Tea; SLAT, Sri Lanka Autumn Tea; SLST, Sri Lanka Spring Tea; SLTB, Sri Lanka Tea Barudi; TI, İndia Tea; TT, Turkey Tea.

Based on the findings, a total of 26 distinct phenolic compounds were identified in tea samples using both qualitative and quantitative analyses. Compounds that exceeded 1000 mg/L were considered to be dominant. Quinic acid, ECG, EGCG, EC, EGC, gallic acid, nicotiflorin, and isoquercitrin were found to be the principal compounds when ranked by their maximum concentration. In addition, the tea samples contained minor components such as quercitrin, hesperidin, astragalin, fumaric acid, catechin, chlorogenic acid, protocatechuic acid, tannic acid, ellagic acid, salicylic acid, p‐coumaric acid, quercetin, kaempferol, and narin. The dominant compound among the established acids was kinic acid, which was present in all tea samples at a rate of 6–9 mg/g dry leaves. Based on our research, gallic acid was found to be the second most dominant organic acid, with the highest amount being present in the sample of Iranian tea (2059 ± 0.12 mg/g of dry leaves).

### 3.2 Results of the determination of Total phenolic compounds

The quantification of the total phenolic content in food is essential to discerning their hydroxyl groups, which offer antioxidant activity. Additionally, as the method extracts extractable proteins present in the environment, it outlines all phenolic groups found within the food's structure. Hence, an unambiguous technique cannot be accepted. A two‐factor analysis was conducted on various tea samples, comprising brewing time and tea origin. The results for the determination of overall phenolic compounds are classified in Table 2. Based on these findings, the brewing duration had a considerable impact on the phenolic content of the tea extracts. With an increase in brewing time, a statistically significant (*p* < .001) rise was observed in the phenolic contents of all extracts, except for the IT and SLST groups, at 30 min. The analysis of tea groups exhibited noteworthy variations in the total phenolic compounds at all brewing durations. The data reveals that factors including tea origin, harvest time, and production technique significantly impact phenolic levels. This finding supports previous studies on black tea found in existing literature (Erturk et al., 2010).

**TABLE 2:** Total phenolic content (mg gallic acid equivalent/g dry plant).

Brewing time	TT	IT	IrT	SLTB	SLAT	SLST
15 min	36.8 ± 2.7^ABCEabc^	45.4 ± 1.1^GHJc^	46.3 ± 2.02^KLMabc^	17.8 ± 1.9^NOabc^	34.3 ± 1.9^Pabc^	57.1 ± 1.8^abc^
30 min	47.3 ± 1.9^ABCEab^	42.7 ± 0.8 ^FGHJb^	54.8 ± 1.1 ^KLMab^	71.5 ± 1.01 ^NOab^	49.4 ± 0.8 ^Pabc^	31.4 ± 1.8 ^ab^
60 min	52.8 ± 2.2^ABCEbc^	69.6 ± 0.9 ^FGHJbc^	89.9 ± 0.9 ^KLMbc^	111.2 ± 4.8 ^NObc^	61.7 ± 2.2 ^Pabc^	98.7 ± 0.8 ^bc^

Abbreviations: IrT, Iranian Tea; IT, İndia Tea; SLAT, Sri Lanka Autumn Tea; SLST, Sri Lanka Spring Tea; SLTB, Sri Lanka Tea Barudi; TT, Turkey Tea. A–P: The mean values with different letters in the same line are significantly different (*p* < .05). a–c: The mean values with different letters in the same column are significantly different (*p* < .05).

### 3.3 Results of the determination of Total flavonoid compounds

A two‐factor analysis was conducted on tea samples, considering brewing time and tea origin. Total flavonoid compounds’ determination results are presented in Table 3, revealing statistically significant differences (*p* < .05) among all tea groups at all brewing times regarding total flavonoid content. The comparison between brewing times illustrated a significant elevation in all tea groups between 15 and 60 min. However, this variation did not show significance among each group after 30 min. Specifically, although an increase in TT, IrT, and SLST groups was anticipated after brewing for 30 min, there was a statistical decrease (*p* < .05) observed instead, which deviated from expectations. Notably, no significant difference was noticed in ABTS radical scavenging activity between 15 and 30 min, but remarkable increases were found after 60 min. These findings indicate that flavonoids play a crucial role in determining the antioxidant capacity of tea.

**TABLE 3:** Total flavonoid compound (mg quercetin equivalent/g dry plant).

Brewing time	TT	IT	IrT	SLTB	SLAT	SLST
15 min	32.9 ± 1.4 ^ACEabc^	18.0 ± 1.4 ^FGJb^	31.6 ± 1.3 ^KLMabc^	10.5 ± 0.5 ^NOabc^	15.1 ± 1.2 ^Pabc^	57.1 ± 0.6 ^abc^
30 min	21.5 ± 2.5^BCDab^	21.6 ± 0.9^FHc^	15.3 ± 1.1^Lab^	17.2 ± 1.5 ^Nab^	22.4 ± 1.4 ^Pab^	15.47 ± 1.6 ^ab^
60 min	41.4 ± 2.5^BCDEbc^	45.8 ± 0.8^GHJb^	48.7 ± 2.1 ^KLMbc^	90.2 ± 3.6 ^NObc^	28.1 ± 1.9 ^Pbc^	71 ± 1.5 ^bc^

Abbreviations: IrT, Iranian Tea; IT, İndia Tea; SLAT, Sri Lanka Autumn Tea; SLST, Sri Lanka Spring Tea; SLTB, Sri Lanka Tea Barudi; TT, Turkey Tea. A–P: The mean values with different letters in the same line are significantly different (*p* < .05). a–c: The mean values with different letters in the same column are significantly different (*p* < .05).

### 3.4 Results of ABTS radical scavenging activity

A 3‐factor analysis was conducted to measure the antioxidant activity in tea samples, considering the concentration difference, brewing time, and origin of tea. Table 4 presents the results of ABTS radical scavenging activity. These results indicate statistically significant increases (*p* ≤ .001) in ABTS radical scavenging activity across all tea samples as the concentration increased. The most significant activity was observed at a concentration of 250 μg/mL in the 60‐min extract of the IT group. Similarly, the highest activity was observed in the SLST group's 60‐min extract at a concentration of 500 μg/mL. Moreover, the tea brewing duration did not have any impact on the antioxidant activity during the first 30 min among the tea groups. While there were noteworthy rises (*p* ≤ .001) at the doses of 250 and 500 μg/mL at 60 min, no significant difference (*p* ≥ .05) was found at the dosage of 1000 μg/mL. Overall, when considering the modification in the source of the tea along with the alterations in the concentration and brewing period, it has become apparent that it does not possess a significant impact on the capacity to prevent oxidation. Increasing the brewing time resulted in heightened antioxidant activity at all concentrations, exclusively in the SLTB tea group.

**TABLE 4:** ABTS activity (μg trolox equivalent/g dry plant).

Tea groups	250 μg/mL			500 μg/mL			1000 μg/mL		
	15 min	30 min	60 min	15 min	30 min	60 min	15 min	30 min	60 min
TT	133.4 ± 2.4	109.8 ± 6.9	112.9 ± 10.6	168.8 ± 6.2	183.1 ± 5.7	187.1 ± 4.9	328.6 ± 6.1	329.9 ± 4.2	329.5 ± 6.7
IT	132.9 ± 2.1	140.9 ± 7.7	149.4 ± 10.1	198.2 ± 5.7	138.7 ± 6.1	193.7 ± 6.1	329.9 ± 4.3	323.7 ± 2.6	322.4 ± 5.2
IrT	132.5 ± 7.7	129.8 ± 12.1	146.3 ± 9.8	203.5 ± 3.5	176.8 ± 5.5	169.7 ± 7.1	330.8 ± 6.8	330.4 ± 3.6	329.5 ± 3.8
SLTB	66.4 ± 4.5	123.6 ± 3.3	137.4 ± 7.3	157.8 ± 5.5	154.2 ± 6.5	195.1 ± 3.4	242.9 ± 4.9	312.2 ± 4.3	330.3 ± 7.3
SLAT	110.5 ± 5.3	109.4 ± 6.9	116.5 ± 8.6	177.7 ± 2.4	157.8 ± 7.6	171.1 ± 6.9	301.9 ± 2.9	297.9 ± 7.3	321.5 ± 6.7
SLST	120.9 ± 3.9	116.5 ± 6.6	133.8 ± 9.5	195.1 ± 1.6	196.8 ± 7.5	250.1 ± 4.3	330.4 ± 5.7	329.9 ± 9.1	330.3 ± 7.4

Abbreviations: IrT, Iranian Tea; IT, İndia Tea; SLAT, Sri Lanka Autumn Tea; SLST, Sri Lanka Spring Tea; SLTB, Sri Lanka Tea Barudi; TT, Turkey Tea.

### 3.5 Results of DPPH radical scavenging activity

A 3‐factor analysis was conducted to measure the DPPH radical scavenging activity in tea samples. The factors studied were the difference in concentration, brewing time, and tea origin. The outcomes of the analysis are presented in Table 5. Statistically significant results were obtained (*p* ≤ .001), indicating that the scavenging activity significantly increased as the concentration increased in all tea samples. The 15‐min extract of the SLST group displayed the greatest activity at the concentration of 250 μg/mL, while the SLST group's 60‐min extract showed the highest activity at the concentration of 500 μg/mL. At a concentration of 1000 μg/mL, the tea groups exhibited activity almost identical to each other. In general, tea brewing times did not result in substantial alterations in antioxidant activity. However, an increase in brewing duration showed a rise in antioxidant activity at all SLTB tea group concentrations. However, at the concentration of 250 μg/mL, the increase in brewing time resulted in significant decreases (*p* ≤ .05) in most groups' antioxidant activity. When considering changes in the tea's origin alongside alterations in concentration and brewing time, it has been largely observed that there is no significant impact on antioxidant activity.

**TABLE 5:** DPPH Activity (μg Trolox equivalent/g dry plant).

Tea groups	250 μg/mL			500 μg/mL			1000 μg/mL		
	15 min	30 min	60 min	15 min	30 min	60 min	15 min	30 min	60 min
TT	95.4 ± 0.9	117.8 ± 3.6	124.4 ± 3.3	214.1 ± 12.1	268.6 ± 14.3	253.4 ± 2.5	359.8 ± 18.7	367.5 ± 6.3	371.1 ± 11.1
IT	122.4 ± 1.3	136.6 ± 2.3	127.9 ± 4.1	218.7 ± 11.8	217.7 ± 12.2	256.9 ± 3.2	368.1 ± 5.9	356.8 ± 5.7	370.1 ± 4.7
IrT	144.8 ± 2.5	89.7 ± 0.9	128.5 ± 5.2	190.6 ± 8.8	261.5 ± 8.5	220.7 ± 6.5	365.5 ± 15.5	325.7 ± 4.2	368.1 ± 6.6
SLTB	123.4 ± 1.7	106.6 ± 2.2	150.9 ± 6.6	129.1 ± 6.9	235.5 ± 14.5	265.6 ± 4.8	249.3 ± 10.2	338.5 ± 6.6	369.1 ± 5.8
SLAT	173.3 ± 5.2	94.8 ± 1.8	137.2 ± 7.3	173.8 ± 11.3	239.6 ± 16.2	201.4 ± 3.6	349.7 ± 11.1	357.3 ± 3.6	366.5 ± 11.4
SLST	184.5 ± 4.6	89.3 ± 10.6	171.8 ± 9.8	239.1 ± 7.8	284.4 ± 8.7	349.7 ± 9.9	369.1 ± 14.3	356.3 ± 2.8	367.5 ± 17.2

Abbreviations: IrT, Iranian Tea; IT, İndia Tea; SLAT, Sri Lanka Autumn Tea; SLST, Sri Lanka Spring Tea; SLTB, Sri Lanka Tea Barudi; TT, Turkey Tea.

### 3.6 Color analysis results

Color is a key factor in the consumer acceptance process. At present, the L*(whiteness/darkness), a*(redness/greenness) and b* (yellowness/blueness) values are widely used as objective criteria for determining color. Table 6 shows the color analysis results of various tea groups after infusion periods of 15, 30, and 60 min.

**TABLE 6:** Color analysis of tea groups (mean ± standard deviation).

Tea groups	L*			a*			b*		
	15 min	30 min	60 min	15 min	30 min	60 min	15 min	30 dk	60 min
TT	7.42 ± 0.33^Aa^	6.73 ± 0.69^Ab^	4.34 ± 0.64^Bd^	−1.16 ± 0.91^Ba^	2.99 ± 1.06^Aa^	3.93 ± 0.64^Ab^	8.29 ± 0.89^Bb^	6.39 ± 1.48^Bb^	11.00 ± 1039^Aab^
IT	6.25 ± 0.79^Bab^	8.20 ± 0.75^Aa^	5.13 ± 0.44^Bc^	−1.61 ± 0.51^Cab^	1.11 ± 0.37^Bc^	2.82 ± 0.37^Abc^	9.28 ± 0.78^Aab^	6.53 ± 0.67^Ab^	5.74 ± 2.97^Ad^
IrT	4.34 ± 0.96^Bc^	5.20 ± 0.52^Bc^	7.92 ± 0.36^Aa^	‐1.2 ± 0.52^Ba^	3.47 ± 0.93^Aa^	2.96 ± 0.81^Abc^	9.30 ± 0.77^Aab^	6.53 ± 1.19^Bb^	8.78 ± 0.79^Abc^
SLTB	5.16 ± 0.52^Cbc^	6.61 ± 0.40^Bb^	7.42 ± 0.10^Aab^	−0.62 ± 0.72^Ba^	3.10 ± 0.81^Aa^	2.49 ± 0.60^Ac^	8.72 ± 1.03^Aab^	4.14 ± 0.84^Bc^	8.54 ± 1.17^Abcd^
SLAT	6.21 ± 0.44^Bab^	8.26 ± 0.60^Aa^	6.68 ± 0.10^Bb^	−1.3 ± 0.17^Ca^	1.46 ± 0.01^Bbc^	2.59 ± 0.64^Ac^	5.85 ± 0.91^Ac^	6.30 ± 0.94^Ab^	6.99 ± 1.01^Acd^
SLST	5.09 ± 0.99^Bbc^	5.11 ± 0.59^Bc^	6.97 ± 0.42^Ab^	−3.33 ± 2.11^Bab^	2.47 ± 0.88^Aab^	3.18 ± 0.26^Abc^	7.40 ± 1.00^Abc^	7.28 ± 0.41^Aab^	7.61 ± 0.99^Acd^

Abbreviations: IrT, Iranian Tea; IT, İndia Tea; SLAT, Sri Lanka Autumn Tea; SLST, Sri Lanka Spring Tea; SLTB, Sri Lanka Tea Barudi; TT, Turkey Tea. A–C: The mean values with different letters in the same line are significantly different (*p* < .05). a–d: The mean values with different letters in the same column are significantly different (*p* < .05).

The L* value is a crucial factor in determining the quality of brewed black tea. The closer the L* value is to zero, the darker and richer the infusion, while a higher L* value results in a brighter infusion. Our analysis revealed statistically significant (*p* < .05) differences between all tea groups when examining the L* value at 15 min. The TT group exhibited the highest L* value (7.42 ± 0.33), while the lowest L* value was observed in the IrT group (4.34 ± 0.96). At the 30‐min mark, there was a statistically significant (*p* < .05) decrease in the TT group's L* value compared to a significant (*p* < .05) increase in the other tea groups. Within the relevant brewing time, the SLAT group had the highest L* value at 8.26 ± 0.60, while the lowest L* value was observed in the SLST group at 5.11 ± 0.59. Upon examination of L* values at the 60‐min mark, it was determined that there was a statistically significant (*p* < .05) reduction in the TT, IT, and SLAT groups. Conversely, there was a significant (*p* < .05) increase in the IrT, SLTB, and SLST groups. Technical term abbreviations such as ‘SLAT’, ‘IrT’, ‘SLTB’, and ‘SLST’ have been explained at their first use. It is noteworthy that biased or subjective evaluations have been deliberately excluded to maintain objectivity. The IrT group had the highest L* value at 60 min (7.92 ± 0.36), while the TT group had the lowest L* value (4.34 ± 0.64). The TT group showed the highest L* value at 15 min, but it subsequently decreased to the lowest L* value at 60 min.

Examining the a* values, which indicate the redness/greenness ratio in the color analysis of food, it was observed that all tea groups had negative values at 15 min. The IT and SLST groups had lower a* values compared to the other tea groups. At 30 min of brewing time, the a* values increased from negative to positive values in all tea groups. At 30 min, the IrT (3.47 ± 0.93), SLTB (3.10 ± 0.81), and TT (2.99 ± 1.06) tea groups exhibited the highest a* value, contrasting with the IT (1.11 ± 0.37) and SLAT (1.46 ± 0.01) groups that yielded the lowest values. However, at 60 min of brewing time, the a* value of the IrT and SLTB groups decreased while that of other tea groups increased. The TT group displayed the highest a* value at 60 min (3.93 ± 0.64). This result demonstrates that an increase in brewing time in the TT tea group leads to a higher red color ratio. The red color, which is a desired characteristic in tea, was more pronounced in the TT group.

Statistically significant differences (*p* < .05) were identified in the b* values, which indicate the yellowness/blueness ratio in the color analyses of food, between various tea groups and brewing times. At the 30‐min brewing mark, there was a statistically significant increase in the b* value of the SLAT group (*p* < .05), with a significant decrease observed in other tea groups. This decrease persisted in the IT tea group at the 60‐min mark, but the b* value increased again in the other tea groups. The highest b* value was observed in the TT (11.00 ± 1.039) tea group after 60 min, whilst the lowest b* value was found in the IT (5.74 ± 2.97) and SLAT (6.99 ± 1.01) groups.

The correlations between the color characteristics, extraction rate, and total phenolic and total flavonoid compounds of tea groups after different brewing times are given in Tables 7 and 8. There was a positively significant (*p* < .05) correlation between total phenolic compound and L* value in the IrT, SLTB, and SLAT groups, but a negative correlation in the TT and IT groups. There was a positive correlation between total phenolic compounds and the a* value in all groups. There was a positive correlation between total phenolic compound and b* value in the groups other than the SLTB group (Table 8). Also, during the brewing time of tea, the correlation between total phenolic compound and L* value was negative at 15 min, positive at 30 and 60 min, and the correlation between total phenolic compound and a* and b* values was positive at 15 min, negative at 30 and 60 min, and the correlation between total phenolic compound and b* value was negatively significant (*p* < .05) at 30 min (Table 8).

**TABLE 7:** Pearson correlation coefficients between total phenolic, total flavonoid, and extraction of color change of tea groups.

Tea groups	Total phenolic			Total flavonoid			Extraction rates		
	L*	a*	b*	L*	a*	b*	L*	a*	b*
TT	−0.884	0.985	0.432	−0.677	0.086	0.982	−0.765	0.999*	0.231
IT	−0.832	0.735	0.603	−0.697	0.861	−0.757	0.254	0.880	−0.951
IrT	0.999*	0.573	0.159	0.738	−0.84	0.754	−0.256	0.610	−0.990
SLTB	0.997*	0.831	−0.122	0.819	0.431	0.402	0.351	0.766	−0.987
SLAT	0.02	0.906	0.997	−0.298	0.724	0.92	0.997*	0.509	0.175
SLST	0.84	0.934	0.582	0.834	0.938	0.573	0.322	0.954	−0.049

Abbreviations: IrT, Iranian Tea; IT, İndia Tea; SLAT, Sri Lanka Autumn Tea; SLST, Sri Lanka Spring Tea; SLTB, Sri Lanka Tea Barudi; TT, Turkey Tea. L* (whiteness/darkness), a* (redness/greenness), b* (yellow/blueness). *p < .05; **p < .01.

**TABLE 8:** Pearson correlation coefficients between total phenolic, total flavonoid, and extraction rate of color change of teas during brewing times.

Brewing time	Total phenolic			Total flavonoid			Extraction rates		
	L*	a*	b*	L*	a*	b*	L*	a*	b*
15 min	−0.343	0.097	0.569	−0.556	−0.4	0.578	−0.370	−0.083	0.241
30 min	0.159	−0.172	−0.796*	0.407	−0.762	−0.048	−0.004	−0.144	−0.608
60 min	0.358	−0.159	−0.13	−0.158	0.312	0.176	−0.111	−0.588	−0.817*

*Note*: L* (whiteness/darkness), a* (redness/greenness), b* (yellow/blueness) *(*p* < .05).

Table 8 demonstrates that a positive correlation existed between the total flavonoid compound and the L* value in the IrT and SLTB groups, whereas a negative correlation was observed in other groups. Furthermore, a positive correlation was found between the total flavonoid compound and a* value in groups other than the IrT group, and finally, a negative correlation was observed between the total flavonoid compound and b* value in groups other than the IT group. During tea brewing, a negative correlation between total flavonoid and L* value was observed at 15 and 60 min, while a positive correlation was observed at 30 min. Additionally, negative correlations between total flavonoid and a* value were found at 15 and 30 min, with a positive correlation at 60 min. Finally, a negative correlation between total flavonoid and b* value was observed at 30 min, with a positive correlation at 15 and 60 min (refer to Table 8 for details).

There was a positive correlation between the extraction rate and the L* value in all groups except for the TT and IrT groups. However, only the SLAT group showed a significant positive correlation (*p* < .05). Similarly, there was a positive correlation between the extraction rate and a* value in all groups, but only the TT group showed a significant positive correlation (*p* < .05). According to the findings of our study, a direct relationship was discovered between brewing time and the extraction rate of the b* value in all tea samples, excluding the TT and SLAT tea groups (as shown in Table 7). Furthermore, there was a negative correlation between the extraction rate and the values of L* and a* during the brewing times of 15, 30, and 60 min. There was a positive correlation between extraction rate and b* value observed only at 15 min and a negative correlation at 30 and 60 min (as displayed in Table 8).

**FIGURE 2:**
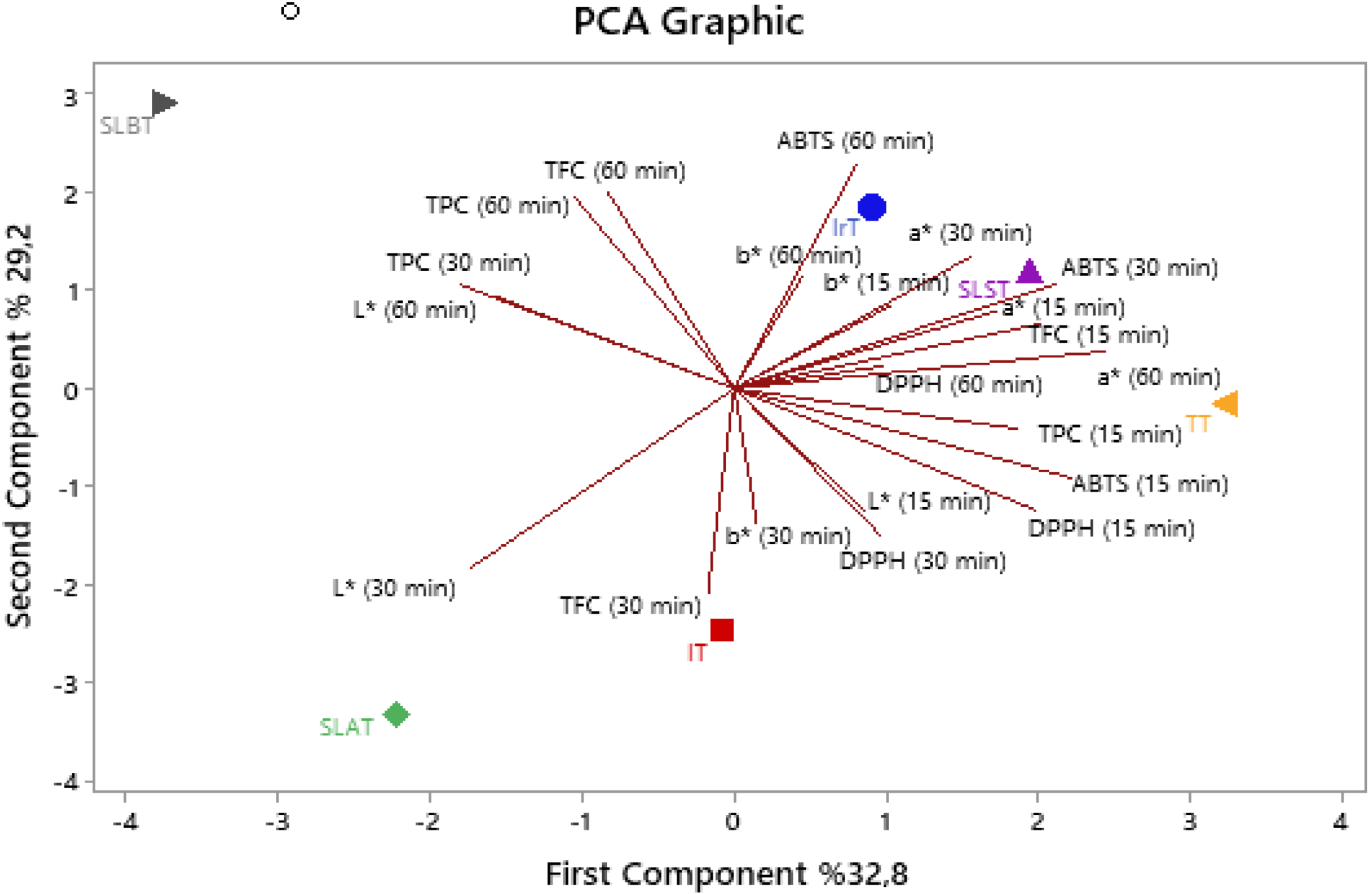
Principal Component Analysis (PCA) graphic. TT: Turkey Tea, TI: İndia Tea, IrT: Iran Tea, SLTB: Sri Lanka Tea Barudi, SLAT: Sri Lanka Autumn Tea, SLAT: Sri Lanka Spring Tea, TPC: Total phenolic content, TFC: Total flavonoid compound, ABTS: 2,2‐Azino‐bis(3‐ ethylbenzothiazoline‐6‐sulfonic acid, DPPH: 1,1‐Diphenyl‐2‐picrylhydrazyl, L*: Whiteness/darkness, a*: Redness/greenness, b*: Yellow/blueness.

PCA data for tea samples were analyzed to examine the correlation between activity and color (L*, a*, b*) values at different brewing times. The results are presented in Figure 2. The PCA analysis indicates that the combined variance accounted for 32.8% of the first component (PC1) and 29.2% of the second component (PC2). The first two principal components (PC1 and PC2) describe 62% of the total data variance, with PC1 exhibiting the highest variance in the data set. The first two principal components (PC1 and PC2) describe 62% of the total data variance, with PC1 exhibiting the highest variance in the data set. Abbreviations used in technical terms are explained in their first instance. Upon analyzing PC1 and PC2 from the tea samples, it can be observed that IrT, SLST, and TT exhibit positive attributes, while SLBT, SLAT, and IT exhibit negative attributes in PC1. Meanwhile, IrT, SLST, TT, and SLBT are characterized as positive, and SLAT and IT are negative in PC2. The most significant bioactive compounds in PC1 are the 15‐min ABTS and DPPH, while TFC and TPC are the most dominant compounds in PC2 after 60 min. It is apparent from the PCA analysis that the color properties and bioactivity vary according to the brewing times for all tea groups. Regarding the color values and antioxidant activity (ABTS and DPPH) properties, SLST, IrT, and TT show a close correlation, with considerably higher results than other teas. The a* value is highest for 15 min SLST and for 60 min TT. Furthermore, the a* values for 30 min IrT and SLT were found to be similar. The highest b* value for IrT was found at 15 and 60 min. As for the L* value, the highest value for 30 min of SLAT was found, whereas SLBT had the highest value at 60 min. There is a negative correlation between the L* value at 30 min and the a* values. The values of a* and b* and the antioxidant activities of ABTS and DPPH show a direct relationship and are related. A relationship exists between the 15‐min TPC and TFC and the a* value. Furthermore, the highest values for TPC and TFC SLBT are observed at 60 min.

## 4 DISCUSSION

The findings from our LC–MS/MS content determination, employing 53 phenolic compounds and applied to tea samples for the first time, are consistent with those of previous literature pertaining to black tea (Akhtar et al., 2020). However, previous studies have yielded conflicting results. Skowron et al. (Magdalena et al., 2018) reported kinic acid as the primary phenolic acid in Chinese‐origin black tea sold in Poland, with a concentration range of 9–11 mg/g of dry leaves determined using LC–MS. Furthermore, the same study found that the average gallic acid concentration in black tea was 1.9 mg/g. Wu et al. (2012) (Wu et al., 2012) conducted an analysis of 8 black tea varieties produced in China, revealing that gallic acid is the predominant phenolic acid in tea, with levels ranging from 0.57 to 1.37 mg/g. Skowron et al. (Magdalena et al., 2018) similarly detected galacic acid in Chinese black tea in comparable amounts. However, our study did not identify any routine compounds in the tea samples. Normally, green tea leaves contain a significant amount of routine compounds (Jiang et al., 2015). However, the routine content is reduced, and the amount of gallic acid in the infusion is increased after fermentation when producing black tea (Jeszka‐Skowron & Zgoła‐Grześkowiak, 2014). The presence of routine compound in Chinese tea (Magdalena et al., 2018) and their absence in other countries’ teas that we examined may be linked to the different fermentation processes.

The phytochemical composition of tea samples cultivated in various countries is comparable in terms of phenolic substances. However, disparities are observed in the quantities of phenolic components. Notably, flavonols display marked differences in volume across different classes and harvesting periods of Sri Lankan tea. The taste and aroma of tea extracts is attributed to specific organic acids present in the plant. These findings may account for the taste variation observed among tea samples from diverse countries and harvesting periods. This is due to the varying quantities of secondary metabolites responsible for the distinct color and aroma of tea, which were found to be present in dissimilar amounts in all evaluated tea samples. For instance, in terms of flavanol compounds, Iranian tea exhibited the highest amount of ECG (5578 ± 0.0012) compared to Turkish tea. Epicatechin content is highest in tea of Iranian origin (4.841 ± 0.001 mg/g dry substance), followed by Sri Lankan tea (3.968 ± 0.0009 mg/g dry substance) and Indian tea (3.701 ± 0.0008 mg/g dry substance). However, it is important to clarify that the amount of flavanol compounds present in a tea is not an indicator of its quality. It should be noted that the Sri Lankan tea was harvested during the summer season. It is simply a variation, and these variations may be the paramount factor in determining the characteristic structure of tea. The most comprehensive research into the phenolic compounds present in Turkish black tea was conducted by Kelebek (Kelebek, 2016), who identified a total of 35 phenolic components. In this present study, we identified a total of 26 phenolic compounds in black teas, utilizing 53 potential phenolic compounds in herbal teas. In contrast to Kelebek's study, our research has identified 15 newly discovered compounds, including nicotiflorin, isoquercitrin, quercitrin, astragalin, fumaric acid, chlorogenic acid, protocatechuic acid, tannic acid, ellagic acid, salicylic acid, p‐coumaric acid, naringenin, luteolin, hesperetin, and apigenin. These findings contribute to the current knowledge of the subject matter.

The quantity of phenolic compounds discovered in tea fluctuates based on several circumstances, including leaf variety, growth environment, time of year, and processing technique. These factors are influenced by climate, culture, and genetics (Hocaoglu, 2007). During the stages of black tea processing, there is a gradual reduction in flavanol content. The oxidation process involved in black tea processing leads to secondary polyphenols such as flavanols and tearubugines, which cause a decrease in flavanol content (Kim et al., 2013). During black tea production, the contents are converted into theaflavin and thearubigins, which provide black tea with its characteristic color and flavor, through a specific process (Samanta et al., 2015). The higher the conversion rate, the lower the primary contents discovered in the tea samples. Consequently, the rate of color imparted by the dry tea is high, and this adversely affects the taste profile (IBM SPSS, 2012; Takım & Işık, 2020). These variations rank highly among the factors that differentiate tea preferences. In fact, teas sourced from Turkey's western regions are favored for their milder taste. Meanwhile, in the eastern regions, despite being at least three times more expensive due to state policies, tea from Sri Lanka is preferred.

The total polyphenol content is significantly related to tea quality parameters. This metric is determined by the genetic makeup of the tea plant and how it is harvested. It has a direct influence on both fermentation time and baking temperature. Extended fermentation periods result in increased levels of thearubigines, which contribute to a combined effect of darker color and a more volatile aroma, yet decreased brightness and aroma index. Conversely, shorter fermentation times lead to the production of black tea with heightened brightness and flavor index (Owuor & Obanda, 1998). Our study reveals that tea in Iran and Turkey contains a high level of flavanols. These compounds are, as previously explained, reduced to theaflavins during the fermentation process. Furthermore, the literature indicates that Turkish tea has low theaflavin levels due to substandard collection standards and poor‐quality fresh tea leaves (Tüfekci & Güner, 1997). We believe that the matter at hand encompasses not solely the tea gathering process in these nations but also the favored variety of tea leaf, the stage of tea collection processing, and the climate conditions of the countries in question (Jeszka‐Skowron & Zgoła‐Grześkowiak, 2014; Kelebek, 2016; Wu et al., 2012). Chinese tea with smaller leaf sizes is produced in Turkey and Iran, while Sri Lanka and India cultivate the larger leafed Assam variety (Pastoriza et al., 2017). It is reported that, especially as the leaf size increases, the surface area of the tea expands and allows better extraction of polyphenols (Almeida et al., 2019). However, in the current study, the direct effect of leaf size on total phenolic content cannot be clearly stated because many other factors are effective. It has been reported that Turkish tea undergoes less fermentation (Hocaoglu, 2007; Jiang et al., 2015; Magdalena et al., 2018). Our findings support the aforementioned reports.

It is reported that the amount of phenolics released increases as the infusion time increases (Gan & Ting, 2019). This is because tea leaves usually swell in order to increase the surface area and facilitate the penetration of water into the leaves. Thus, the longer the infusion time, the more the tea leaves swell and the more surface area is achieved, resulting in more phenolics being released into the water. The results of the present study support this information. It was observed that the total amount of phenolic compounds increased when the brewing time increased from 15 min to 60 min in all tea groups (Table 2). However, this study showed that 15 and 30 min infusion time may be the optimum infusion times to obtain high phenolic levels and that a longer infusion time may not be necessary and may not provide additional benefits. Because it is seen that the antioxidant properties of teas decrease during a 60‐min infusion. Other studies on this subject support this finding (Fernando & Soysa, 2015b; Gan & Ting, 2019).

The LC–MS/MS results for tea samples reveal a clear correlation with both phenolic content and total phenolic compound analysis. Notably, the Sri Lankan Barudi Tea (SLTB) group appears to possess the highest levels of total phenolic compounds. Similarly, the LC–MS/MS results indicate that the SLTB group has the highest content of quinic acid, fumaric acid, and gallic acid among all the tea groups, which have the highest amount of phenolic compounds. This correlation demonstrates the mutual support of our analyses and their accuracy. The levels of total phenolic compounds found in this study align with those reported in the literature (Pereira et al., 2014) (Kelebek, 2016).

ABTS and DPPH activity results correlated favorably, indicating that tea originating from Turkey and Iran had the highest antioxidant activity. This might be due to the abundance of phenolic compounds, including quinic acid, fumaric acid, and gallic acid, revealed by LC–MS/MS analysis, when compared to other tea groups. The variation in the L*, a*, and b* values among tea groups can be attributed to the rise or fall in the phytochemical compounds of the infusion, relying on the brewing time and particle size of tea leaves. Yu et al. (2021) obtained corroborating outcomes in their investigation.

Color analysis revealed significant differences (*p* < .05) in color between the different tea groups, with higher levels of total flavonoid compounds resulting in a stronger infusion and a darker red hue. These findings suggest a direct correlation between the phenolic and flavonoid content and tea's sensory characteristics. The duration of the infusion impacts the extraction of catechins. As the infusion duration increases, a greater quantity of bioactive compounds are extracted (Yu et al., 2021). Our study obtained supporting findings. The extraction rates of the tea groups increased along with the brewing times and the number of total flavonoid compounds with the extraction rates, which affected the color of the tea groups and led to an increase in red color.

It has been reported in the literature that flavonoids and theaflavins constitute the primary compounds responsible for the color of tea extractions (Owuor & Obanda, 1998). Based on our findings, Sri Lankan barudi tea (SLTB) is the top‐performing tea group in terms of total flavonoid compounds. The LC–MS/MS results showed that the SLTB group contains the highest number of flavonoids, which is further supported by the color analysis indicating the highest value in terms of red/green color is also found in the SLTB group. These two sets of results have mutually confirmed each other, providing strong evidence for the superiority of the SLTB group.

The values of black tea collected and reported by (Zhang et al., 2013) in China were similar to the color levels we had measured in tea samples. Salman et al. (Ozdemir et al., 2019) reported that the relevant brewing time (15, 20, 25 min) had no effect on the color and brightness of the Turkish Tea group. However, we observed in our study that the brewing time resulted in changes in the color of the Turkish Tea group. On the other hand, Cagindi and Otles (Çağındı, 2008) reported that there were changes in the L* and a* values of the Turkish tea group during the brewing time, but there was no change in the b* value, which was similar to our results. Laddi et al. (Laddi et al., 2013) ‘s study on the determination of the quality parameters of the Indian tea group reported that an increase in the a* value and a decrease in the b* value of the Indian black tea group were indicators of quality tea. Based on our study results, it was found that there was an increase in the a* value of the Indian tea group but a decrease in the b* value during the brewing time. However, this property was not observed in other tea groups. Dmowski et al. (Dmowski et al., 2014) reported the a* values in the Indian tea group and the b* values in the Turkish tea groups; these values were like our findings, while other values were observed to be higher than the values we found.

## 5 CONCLUSSION

A study was conducted that identified the phytochemical compounds present in black tea from Turkey, India, Iran, and Sri Lanka. The 53‐item phenolic compound method was employed, which was found to be the most advanced approach in this field and was validated by the researchers. The study also examined how the quality characteristics of the tea samples varied during different brewing times. The findings revealed that Turkish and Persian tea had the highest phytochemical content and antioxidant activity among all the samples tested. The red color of tea, as a sensory attribute, was found to be more pronounced in the Turkish tea group. Prolonged brewing time resulted in a substantial increase in infusion color, antioxidant activity, and the total phenolic and flavonoid content. While flavonoids in tea are crucial antioxidant compounds for promoting good health, excessive amounts can have a prooxidant effect and be detrimental to health. Extending the brewing time of tea has been found to have favorable effects on the infusion, including increased infusion amount, improved infusion color, and higher total phenolic and flavonoid contents. However, it is not advised to exceed a brewing time of 60 min, as prolonged brewing may result in adverse health effects.

## AUTHOR CONTRIBUTIONS

**Mehmet Emin Aydemir:** Conceptualization (equal); data curation (equal); formal analysis (equal); resources (equal); software (equal); writing – original draft (equal); writing – review and editing (equal). **Kasım Takım:** Data curation (equal); formal analysis (equal); software (equal); writing – original draft (equal); writing – review and editing (equal). **Mustafa Abdullah Yılmaz:** Conceptualization (equal); formal analysis (equal); writing – original draft (equal).

## CONFLICT OF INTEREST STATEMENT

The authors declare no conflict of interest relevant to this article.

## ETHICS STATEMENT

This article does not cover any human or animal studies conducted by any of the authors. Not applicable.

## Supporting information


Table S1.

## Data Availability

The data are available upon request from the authors.
